# Influence of Acute High Glucose on Protein Abundance Changes in Murine Glomerular Mesangial Cells

**DOI:** 10.1155/2016/3537863

**Published:** 2015-12-29

**Authors:** Michelle T. Barati, James C. Gould, Sarah A. Salyer, Susan Isaacs, Daniel W. Wilkey, Michael L. Merchant

**Affiliations:** ^1^Kidney Disease Program, Department of Medicine, University of Louisville, Louisville, KY 40202, USA; ^2^Harvard Medical School, Boston, MA 02115, USA; ^3^Tuskegee University School of Veterinary Medicine, Tuskegee, AL 36088, USA

## Abstract

The effects of acute exposure to high glucose levels as experienced by glomerular mesangial cells in postprandial conditions and states such as in prediabetes were investigated using proteomic methods. Two-dimensional gel electrophoresis and matrix assisted laser desorption ionization time of flight mass spectrometry methods were used to identify protein expression patterns in immortalized rat mesangial cells altered by 2 h high glucose (HG) growth conditions as compared to isoosmotic/normal glucose control (NG^⁎^) conditions. Unique protein expression changes at 2 h HG treatment were measured for 51 protein spots. These proteins could be broadly grouped into two categories: (1) proteins involved in cell survival/cell signaling and (2) proteins involved in stress response. Immunoblot experiments for a protein belonging to both categories, prohibitin (PHB), supported a trend for increased total expression as well as significant increases in an acidic PHB isoform. Additional studies confirmed the regulation of proteasomal subunit alpha-type 2 and the endoplasmic reticulum chaperone and oxidoreductase PDI (protein disulfide isomerase), suggesting altered ER protein folding capacity and proteasomal function in response to acute HG. We conclude that short term high glucose induces subtle changes in protein abundances suggesting posttranslational modifications and regulation of pathways involved in proteostasis.

## 1. Introduction

Renal glomerular mesangial cells (GMCs) functions are altered in diabetic nephropathy by chronic exposure to high glucose (HG) or exposure to glycated albumin [1–4]. The early effects of hyperglycemia are thought to be dominated by hemodynamic factors including glomerular hyperfiltration and shear stress leading to damage by microalbuminuria or proteinuria [5–10]. The early histopathology of diabetic nephropathy is characterized by a thickening of the glomerular basement membrane (GBM) and an accumulation of extracellular matrix (ECM) in the glomerular mesangium. The damaging effects of chronic hyperglycemia on various kidney glomerular cell types such as mesangial cells, podocytes, and endothelial cells have been intensely studied. The theories that have been addressed include increased substrate channeling into the polyol pathway and the hexosamine pathways and increased production of reactive oxygen species (ROS) and activation of protein kinase C (via advanced glycation end-products (AGE), diacylglycerols (DAG), and/or reactive oxygen species (ROS)) [11–13]. These advances in our understanding of the effects of chronic hyperglycemia on renal physiology have not been matched by understanding of the effects of acute (2 h) hyperglycemic conditions episodically experienced by cells like the GMC in states such as prediabetes. We hypothesize that understanding these acute changes induced by hyperglycemia might yield insight into the mechanisms through which chronic hyperglycemia disrupts mechanisms used to maintain normal glomerular function.

## 2. Material and Methods

### 2.1. Cell Culture

The rat GMC line CRL-2573 (ATCC) maintained normal growth media (DMEM: 5 mM D-glucose, 15% FBS) under 5% CO_2_ at 37°C. The cells (passages 10–15) were plated in Corning T25 flasks and cultured until 70–80% confluence was reached. Normal media were removed from cells and replaced with DMEM supplemented with 0.5% FBS/5 mM D-glucose. After 24 h, media were removed and replaced with isoosmotic-normal glucose (NG^*∗*^) media (DMEM-5 mM D-glucose, 20 mM mannitol, and 0.5% FBS) or high glucose (HG) media (DMEM: 25 mM D-glucose, 0 mM mannitol, and 0.5% FBS), for 2 h. For 2DE analysis, after 2 h treatment, the total protein was collected as previously described [14] using IPG rehydration buffer supplemented with protease inhibitors.

### 2.2. Cell Viability

Cell viability was determined after 2 h HG and NG^*∗*^ treatment using the MTT assay [15] as described by the manufacturer (Sigma, St. Louis, MO, USA).

### 2.3. Two-Dimensional Electrophoresis (2DE) and Image Acquisition

2DE experiments were conducted as reported previously [14]. Murine GMC protein (75 *μ*g) was rehydrated overnight into IPG (pH 3–10; 7 cm; Invitrogen) strips. The strip was focused for a total of 1200–1300 Vh with a final 30 min focusing period at 2000 V constant. Proteins were separated in the second dimension on 4–12% Bis-Tris mini gels (8 cm × 8 cm). The gel slabs were fixed in 10% methanol and 7% acetic acid and then transferred to SYPRO-Ruby protein gel stain (Molecular Probes, Oregon, USA) for 18 hours. Gels were scanned using a PerkinElmer ProXpress CCD-based digital imager at 50 *μ*m resolution. The gel/stain exposure and emission acquisition times were varied to maximize the detector response while avoiding detector saturation. The image files were matched, reference gels were created, and spot volumes were determined using Progenesis Discovery software (Nonlinear Dynamics, Newcastle upon Tyne, UK). A student's *t*-test is used to evaluate all matched spot pairs. Protein spots that were found to have variable spot volumes between samples were statistically compared by spot mean and SEM.

### 2.4. Proteomic Analyses

Protein gel spots were digested as previously described [14]. MALDI-TOF and TOF/TOF MS data were acquired on the tryptic digests using an AB4700 Proteomics Analyzer (Applied Biosystems, Foster City, CA) and analyzed using Matrix Science Mascot (ver. 2.0) as described previously [16]. Data was analyzed assuming (a) monoisotopic peptides masses, (b) cysteine carbamidomethylation, (c) variable oxidation of methionine, (d) maximum of one missed trypsin cleavage, and (e) a mass accuracy of greater than 150 ppm for MS data and 0.3 Da for MS-MS data against the SwissProt (release 52.0, 20070307) protein database (261513 sequences; 95638062 residues) constrained to the mammalian (50870 sequences) taxa. Limitation of the original protein mass was not employed within the Mascot search. Protein identifications were accepted for protein identifications that include using MASCOT MS + MS/MS analysis with significant MOWSE scores (*p* < 0.05; for MS MOWSE score of 60 which equals significance and for MS/MS MOWSE peptide ion score alone of 40 which equals significance).

### 2.5. Confocal Microscopy

Confocal microscopy images were obtained as previously described [17]. Briefly, multichambered cover glass wells (Nunc, Naperville, CT) were seeded with GMC cells. Cells were serum starved with 0.5% FBS-NG medium 24 h before 2 h glucose treatment. Cells were rinsed three times with PBS that contained calcium and magnesium and fixed in 3.7% paraformaldehyde in PBS for 10 min, followed by permeabilization with 0.025% NP-40 in PBS for 15 min. Cells were incubated with primary antibody (1 : 250 anti-PHB in PBS/0.025% NP-40) at 20°C, rinsed five times with PBS/0.025% NP-40, and incubated with the Alexa Fluor 488 conjugated secondary antibody (1 : 1000) at 20°C. The cells were rinsed five times with PBS/0.025% NP-40, incubated with 300 nM DAPI for 5 min, and rinsed three times with PBS. Images were acquired using a Zeiss confocal microscope and analyzed using LSM510 software. Z scan analysis was performed by scanning at 1 *μ*m intervals and three-dimensional reconstruction of the fluorescence images. The images for PHB and for DAPI were merged in a single image to elucidate PHB cellular distribution. Fluorescence intensity measurements (mean fluorescence intensity per *μ*m^2^) were computed per cell (*n* = 4-5 cells per treatment replicate per treatment condition) and used to estimate differences in PHB nuclear and cytoplasmic distribution.

### 2.6. Protein Immunoblotting (IB)

1DE and 2DE protein immunoblots (IB) were conducted as previously described [14]. Total cell lysate samples were separated by 2DE (*n* = 3 HG, *n* = 3  NG^*∗*^). For 2DE IB analysis, following IEF of mesangial proteins, the plastic backing of the IPG strips was trimmed off. The acidic most point of the strip was aligned in the IPG well of the Bis-Tris mini gels adjacent to the MW standard lane of the minigel. This procedure insured uniform alignment of IPG strips to the MW standards, in order to compare PHB migration pattern between experimental conditions. Following 1DE or 2DE electrophoresis and transfer, membranes were immunoblotted for PHB (Santa Cruz Biotechnologies, Santa Cruz, CA) at a 1 : 1000 dilution in 5% albumin in Tris-Tween-20 buffered saline (TTBS). PHB spots were imaged on film with luminol images aligned and quantified by densitometry analysis comparing the means of the acidic third and basic third of the PHB charge trains to the total train densitometry. Additional antibodies used for 1DE immunoblots were anti PDI (Stressgen; San Diego, CA) at a concentration of 1 : 10,000 and PSMA2 (Cell Signaling; Danvers, MA) at a concentration of 1 : 1000.

### 2.7. Analysis of Protein Expressional Networks

Ingenuity Pathways Analysis bioinformatic tool (Ingenuity Systems, Mountain View, CA) uses a curated database (Ingenuity Systems Knowledge Base) of previously published findings on mammalian biology from the public literature to evaluate proteins lists inclusive of expression ratios for protein expressional patterns. The purpose of the evaluation is to establish within the lists of provided expressional data relational networks of protein interactions (e.g., direct protein-protein interaction and transcriptional control). Analysis of submitted protein lists with expressional ratios using the Ingenuity knowledge base was used to identify direct interactions between mammalian orthologs.

Murine GMC proteins demonstrating statistically significant expression between 2 h HG and 2 h NG^*∗*^ as well were analyzed by the Ingenuity Knowledge Base and Pathways Analysis tool. The data output identifies nodes characterizing individual proteins and edges characterizing biological relationships. Putative protein networks are rank ordered according to *p* value (−log_10_^*p*^⁡ ), where the *p* value is a measure of random association of the listed proteins.

### 2.8. Statistical Analysis

Statistical analysis of relative spot pixel intensity from 2D gels (*n* = 5, 2 h each group) and analysis of PHB, PDI, or PSMA2 for HG versus NG^*∗*^ expression by IB was performed using two-tailed, unpaired *t*-test. *p* values < 0.05 were considered significant.

## 3. Results

### 3.1. Alteration of Protein Expression by Acute High Glucose

**Figure 1:**
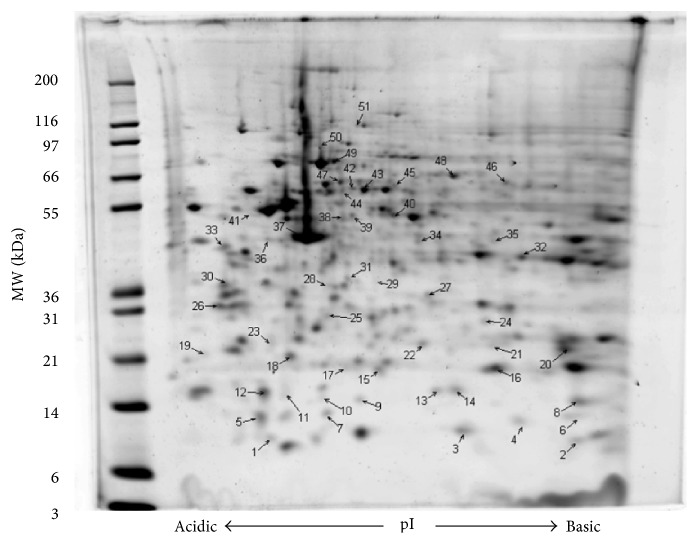
Murine GMC proteome altered by acute (2 h) exposure to HG culture conditions. GMC cells were grown to 80% confluence and were serum-starved (0.5% FBS) overnight, and were treated for 2 h with 25 mM glucose (HG) or 5 mM glucose + 20 mM mannitol (NG^*∗*^) as an isoosmotic control. Cells were lysed using 2DE buffer and 75 *μ*g protein used for 2DE analysis. Proteins whose expression is altered by 2 h HG are annotated on the gel with identifications provided in Table 1. Data are representative of five individual gels for HG and for NG^*∗*^ conditions.

Based on the MTT assay results (data not shown), GMC viability did not statistically vary between 2 h HG and NG^*∗*^ treatments. To determine proteins regulated by 2 h HG treatment, protein spot volume lists were curated by first estimating intergel variability in matched protein spot volumes (averaged CV for 20 matched spots = 0.17). Next, all intraglucose treatment matched gel spot volumes having a CV greater than 0.35 (2 × CV) were discarded. Fifty-one (51) protein spots had a spot volume CV of less than 0.35 and uncorrected *t*-test values of ≤0.05. Thirty-five protein spots had increased expression and 16 protein spots had decreased expression with 2 h HG treatment and all were analyzed using proteomic methods based on MASCOT MOWSE scoring including MALDI TOF/TOF peptide fragmentation (sequence tagging) data with a significance *p* value ≤ 0.05 for all the reported protein identities. A representative 2DE gel image (with annotations) and tabulated information for 51 regulated protein spots are provided (Figure 1; Table 1). In general, all proteins identified were observed migrating in the gels at the correct molecular weight plus or minus 10% except for gel spot 5. Cofilin-1 was identified migrating at a molecular weight of approximately 9000 Da and a pI of 5.3. Cofilin-1 nominally has a translated molecular weight of 18,749 Da and a pI of 8.2. Two additional cofilin-1 containing gel spots as well as one HSP10 containing gel spot were observed to focus on isoelectric points less than 0.5 pH units, more acidic than expected. Twenty-three proteins were observed to focus on isoelectric points greater than 0.5 pH units, more basic than expected. The remaining gel spots identified proteins within 0.5 pH units of the expected pI.

### 3.2. Analysis of Protein Expressional Networks

**Figure 2:**
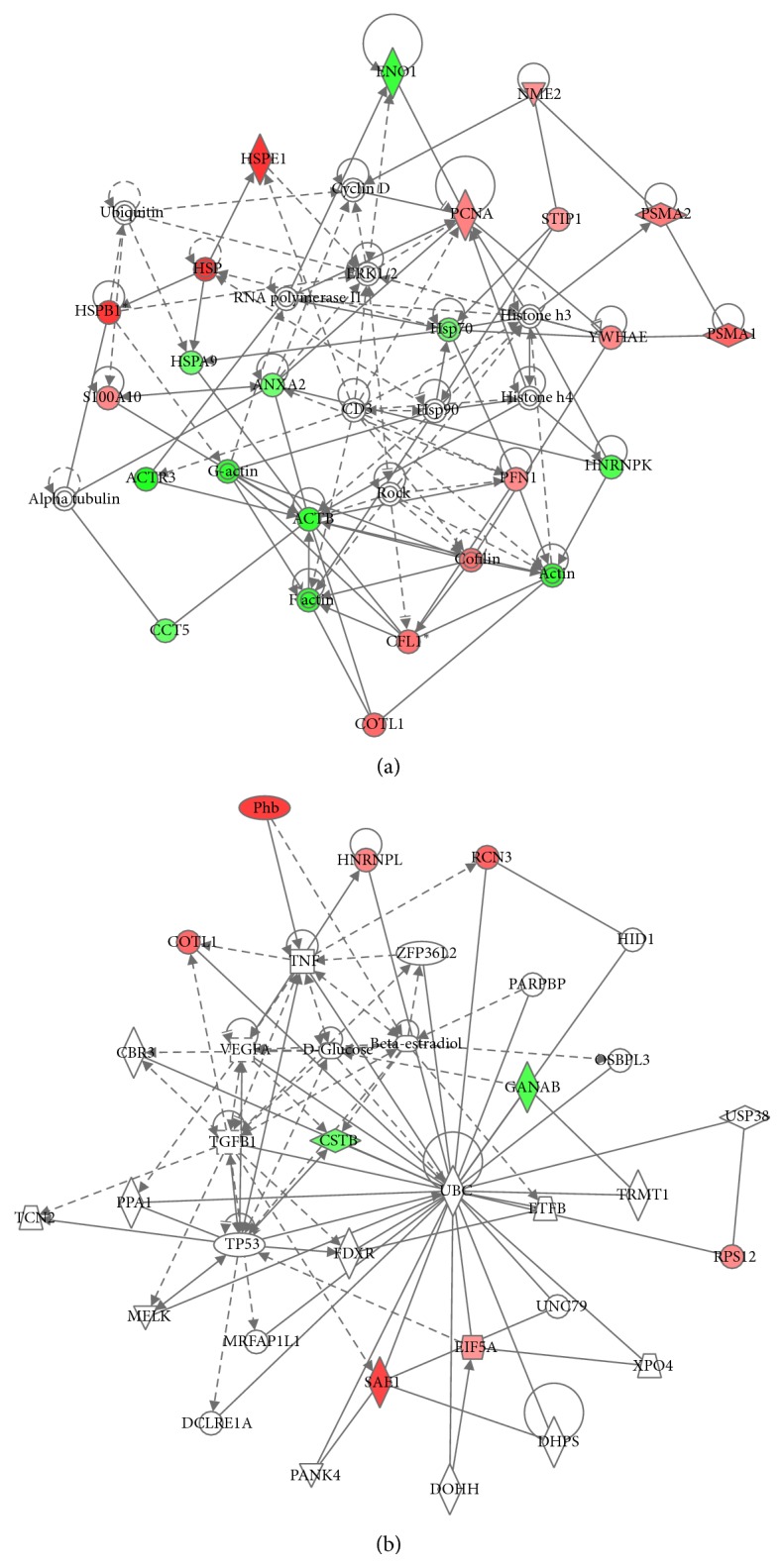
Network analysis of protein expression patterns using Ingenuity Pathways Analysis. (a) The top scoring network (Network 1) addressed cancer, reproductive system disease, and hematological disease and included 25 identified proteins out of 35 total network components. The score 49 suggests the odds of 1 out of 10^49^ for assembling randomly these protein identifications out of the existing murine protein database. (b) Network 2, defined by IPA, includes PHB. For (a) and (b), red indicates protein spots whose spot volume increased with 2 h high glucose. Green indicates proteins spots whose spot volume decreased with 2 h high glucose.

Bioinformatic analysis of protein expression in 2 h NG^*∗*^ versus 2 h HG was achieved using the Ingenuity Knowledge Base and Pathways Analysis tools. The top three canonical pathways determined to be activated from 2 h acute high glucose exposure were actin-based motility by Rho, RhoA signaling, and the protein ubiquitination pathway. Analysis of protein expressional networks from murine GMC 2 h NG^*∗*^ and 2 h HG protein expressional data suggested three primary expression networks. Network 1 (score 49) addressed cancer, reproductive system disease, and hematological disease and included 25 identified proteins out of 35 total network components (Figure 2(a)). Network 2 (score 19) addressed cell death and survival, drug metabolism, and lipid metabolism and included 9 identified proteins out of 35 protein nodes (Figure 2(b)). Network 3 (score 14) addressed cellular movement, cellular compromise, cellular function, and maintenance and was composed of 7 identified proteins out of 29 possible network proteins. Prominent nodes within Network 1 were centered on signaling proteins including proteins involved with ubiquitination, cyclin D, ERK1/ERK2 MAPKinase, HSP90, ROCK, and histones h3 and h4. Prominent nodes in network 2 were centered on the VEGF, TNF, TGF*β*1, tumor protein 53 (TP53), and ubiquitination.

### 3.3. Immunochemical Analysis for the Effect of High Glucose on the Expression of Proteins

**Figure 3:**
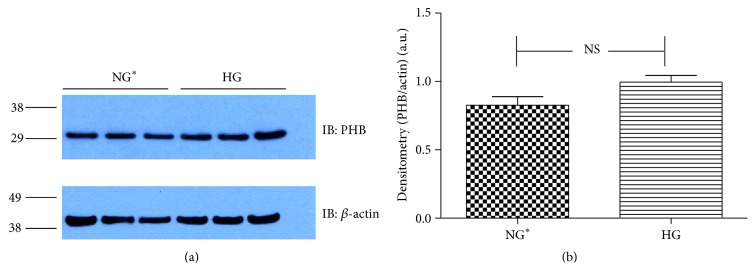
Validation of 2DE results for the enhanced PHB expression. Murine GMCs were cultured and treated for 2 h with HG and NG^*∗*^ as described. Cells were lysed in 2DE buffer, diluted into Laemmli buffer, and used for immunoblot experiments (a) and quantification of 1DE IB experiments for PHB expression normalized to total actin expression (b). Data is presented as a mean of three experiments. Statistical analysis of differences between the means of HG and NG^*∗*^ was achieved by *t*-test.

**Figure 4:**
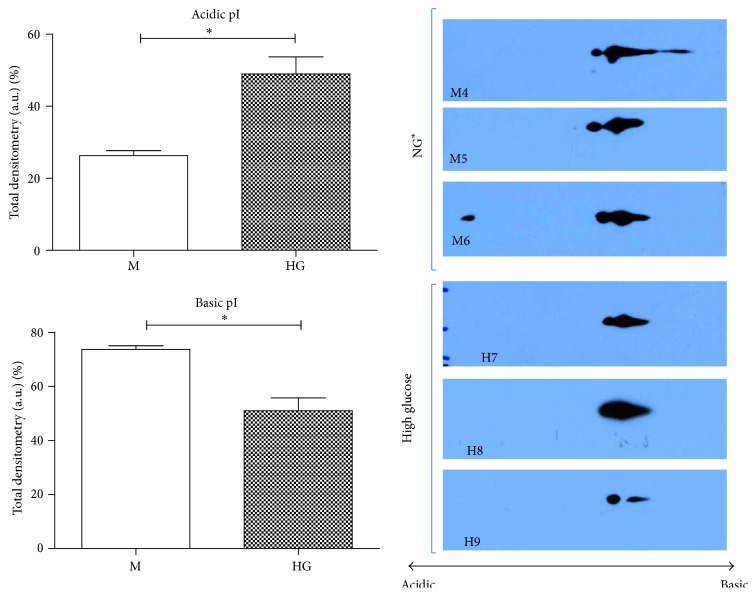
2DE immunoblot experiments were used to determine the effects of HG and NG^*∗*^ on PHB isoforms. Following the transfer and development of PHB IB, images were aligned and densitometric measurements were estimated using ImageJ for the acidic one-third of the PHB charge train and for the basic two-thirds of the PHB charge train (IB images on right). Fractional values for PHB charge train components (acid and basic ends) were used to determine statistical significance differences (left bar graphs). M is the same as NG^*∗*^ (5 mM D-glucose + mannitol); HG is 25 mM D-glucose. ^*∗*^ *p* value < 0.01.

**Figure 5:**
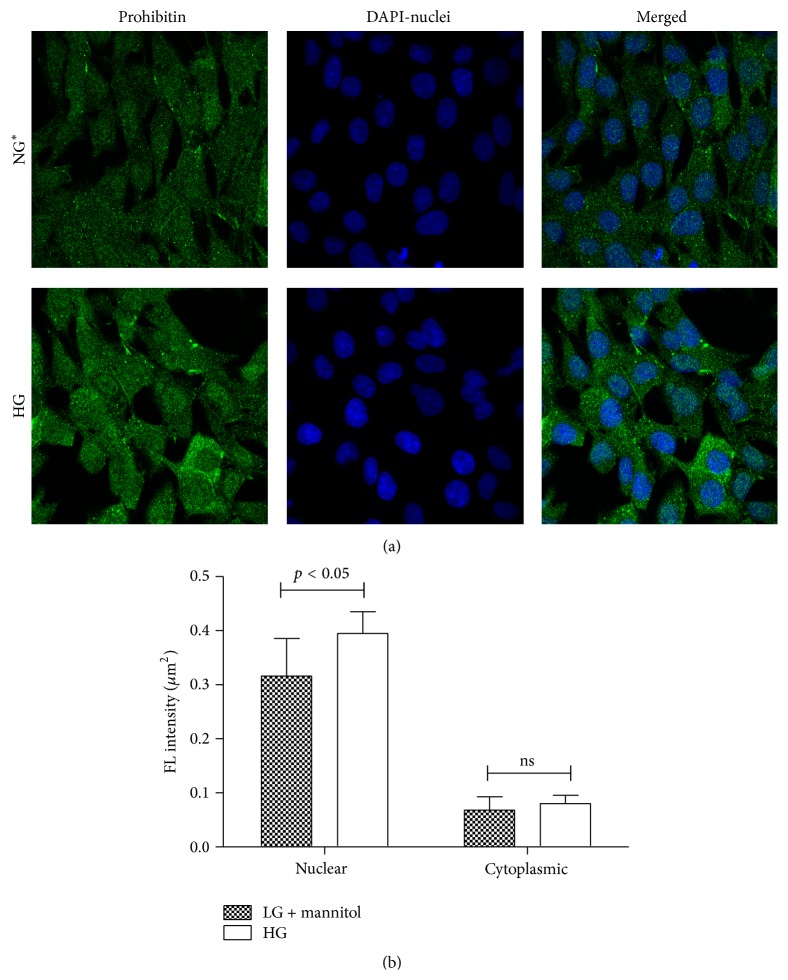
(a) Murine GMCs were seeded into 8-well chambered cover glass, grown, and treated as described in the methods. PHB detection was with the same primary antibody as used for IB. PHB detection with an Alexa Fluor 488 conjugated secondary antibody (green). Nuclei were stained with DAPI (blue). Confocal software was used to estimate pixel density in GMC and in nuclei (as defined by DAPI). Nuclei pixel density was subtracted from total density and plotted (b). Differences were estimated by *t*-test with significance at *p* value < 0.05.

Immunoblot (IB) analyses of the selected proteins were used to confirm the 2DE findings. Prohibitin (PHB) was selected for confirmation as it was one of the most strongly regulated protein spots and was also a component of IPA Network 2 with direct interaction with a prominent network node of TNF. The expression by 1DE (Figures 3(a) and 3(b)) supported a trend in increased total PHB abundance, but 2DE IB analysis of 2 h GMC cells cultured in HG and NG^*∗*^ showed a HG responsive and statistically significant (*p* < 0.02) increase in the acidic end of the PHB charge train (Figure 4). Confocal microscopy (Figures 5(a) and 5(b)) suggested that high glucose resulted in a statistically significant (*p* value < 0.0001) increased fractional abundance of PHB in the nucleus of the GMC.

**Figure 6:**
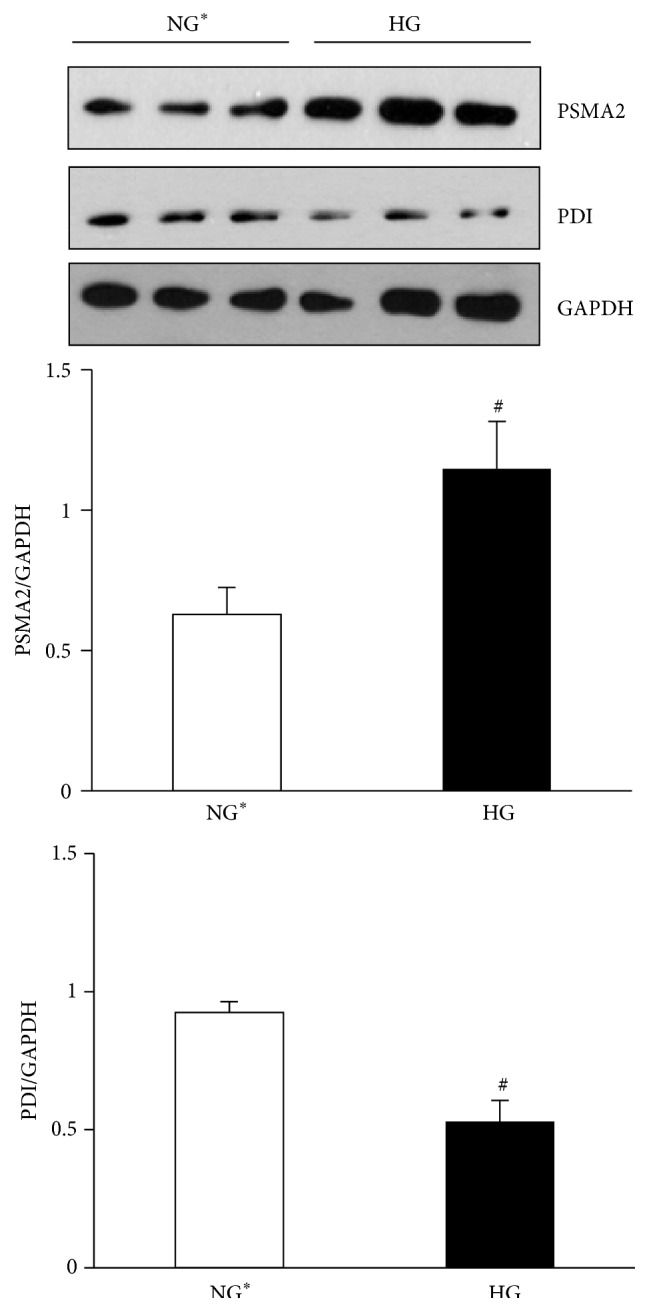
Correlative validation of 2DE results for the regulation of pathways involved in proteostasis. Immunoblot analysis of PSMA2 and PDI from mesangial cells cultured for 2 h in HG or NG^*∗*^ medium. Expression of PSMA2 increased whereas PDI decreased following 2 h HG. Bar graphs, densitometric quantitation of PSMA2 or PDI normalized to GAPDH for each lane. Data are average ± SEM ^#^ *p* < 0.05 versus NG^*∗*^.

Based on bioinformatics analysis defining regulation of protein ubiquitination pathways in one of the top three canonical pathways regulated, as well as a prominent node in protein expression Networks 1 and 2, we next analyzed the expression of proteins involved in protein homeostasis and found them to be regulated by 2DE analysis. Proteasome subunit alpha-type 2 (PSMA2) was confirmed to be increased in mesangial cells exposed to high glucose concentrations for 2 h (Figure 6). Comparative 2DE analysis also defined decreased expression of ER chaperone proteins such as PDI and GRP58, which may lead to increased unfolded protein load in mesangial cells and induction of proteasomal degradation processes. Immunoblot analysis of mesangial proteins for PDI confirmed 2DE findings of decreased expression of PDI (Figure 6).

## 4. Discussion

GMCs participate in glomerular growth and differentiation as well as in regulation of glomerular blood flow [3, 4]. It is well established that chronic hyperglycemia such as in an uncontrolled diabetic state detrimentally affects the renal glomerulus and produces a pathologic GMC phenotype [18–22]. On the other hand, a gap in knowledge exists for changes in GMC function and protein expression patterns which occur in individuals who experience longer postprandial elevated plasma glucose levels [23]. Therefore to ascertain the effects of short term high glucose conditions encountered by GMC in subpathologic/prediabetic states, we conducted proteomic studies comparing mesangial protein expression after 2 h HG conditions against mesangial protein expression after 2 h NG^*∗*^ growth conditions. The analysis of cell viability at 2 h in the treatment conditions determined that mesangial cell viability was not decreased by the treatment conditions and time. The protein expression differences observed between the growth conditions were not therefore attributed to variable degrees of cell proliferation. Expressional regulation of 51 identified protein spots were observed under the conditions of 2 h HG. These proteins can be grouped as follows: cytoskeletal proteins, calcium/phospholipid binding proteins, chaperones, and proliferation and signaling-related proteins.

Increased glucose levels are known to stimulate a variety of responses within GMC including remodeling of cytoskeletal elements like actin and actin binding proteins [24]. Two upregulated spots were identified as cofilin-1 and cofilin-2 and demonstrated a 55–60% increased expression. Cofilins are actin binding proteins that affect the mobility of actin monomers at the ends of actively growing actin filaments and increase actin filament turnover. Cofilins bind and sever the pointed actin ends and increase the actin monomer pool. During conditions of stress, cofilins participate in the nuclear import of actin [24]. Two protein spots demonstrating reduced expression by HG, identified as the actin capping proteins, F-actin capping protein *β*-subunit, and actin-like protein 3. Each of these proteins migrated at the expected *M*_*r*_ and pI. These proteins, respectively, demonstrated a 30% and 50% decreased presence in the 2DE gels. Additionally, an acidic isoform of the intermediate filament protein vimentin was downregulated.

Calpactin light chain (also referred to as S100A10 or p11) functions as a ligand of annexin II (annexin II_2_ : p11_2_) [25–27]. Calpactin and annexin II were shown here to be upregulated by approximately 37% and 23%, respectively by acute hyperglycemic conditions. Calpactin complexed to annexin II is known to interact with the C-terminus of cytosolic phospholipase A2 and inhibits cPLA2 activity thus reducing inflammatory responses from the release of arachidonic acid [28]. Upregulation of reticulocalbindin 3 is necessary for increased sequestration of Ca^2+^. The increased Ca^2+^ is in turn needed by other proteins found in the reticuloplasm like GRP78 or PDIA3 [29]. These observations of regulated changes in actin cytoskeletal protein and calcium binding protein expression, when taken together, are consistent with the known responses of mesangial cells to HG under more chronic conditions [30, 31].

Molecular chaperones have been well described in the literature as protein quality control managers that assist with the maintenance of cellular function in the face of stress conditions like heat stress, osmotic stress, or oxidant stress. Specific chaperones are spatially organized throughout the cell via organellar localization [32, 33]. The bulk of all mitochondrial proteins are synthesized under the direction of cell nuclear transcripts in the cytoplasm [34]. High molecular weight proteins are trafficked through and between the mitochondrial membranes and into mitochondrial matrix and require protein folding chaperone such as PHB and HSP10 for efficient protein folding [35]. A protein spot containing PHB, possibly a posttranslationally modified form causing an acidic shift in PHB pI, was found to exhibit expressional regulation by 2DE, 2DE IB and increased nuclear localization by confocal microscopy analysis, following acute (2 h) glucose exposure in GMCs. PHB has been reported to exist as a membrane resident chaperone that participates in the protein folding pathway of mitochondrial-derived integral membrane proteins like COX2p and COX3p. Moreover, movement of PHB between the mitochondria and nucleus has been shown to play an important role in signaling mitochondrial oxidant stress and regulating apoptosis and transcription during stress, highlighting the importance of this protein to mitochondrial-nuclear communication [36, 37]. In the current study, observations of increased acidic forms of PHB, increased PHB nuclear localization, increased HSP10, and decreased GRP75 at 2 h HG stimulation suggest that acute hyperglycemic conditions may promote protein-structural stress within the mitochondrial matrix promoting translocation of PHB to the nucleus for an as-of-yet determined reason in GMCs. In addition, bioinformatic analysis grouped PHB and additional proteins regulated by 2 h HG in a network including mediators known to be involved in the pathogenesis of diabetic nephropathy and fibrosis, such as TGF*β*, VEGF, and TNF [1, 38], highlighting a potentially novel role for PHB in GMC responses to HG.

One aspect of cell cycle control is polyubiquitination of cytoplasmic or nuclear proteins [39]. Polyubiquitination is a trigger for the trafficking of the modified protein to the proteasome for degradation. A second aspect of cell cycle control is exercised through monoubiquitination of nuclear proteins like histones [40–42]. Our observations with increased expression of PSMA2 are specific to acute exposure of cells to medium containing high glucose as compared to isoosmotic low glucose medium and suggest the likelihood of increased proteasomal activity. These findings are in part supported by the observations of decreased ubiquitinated cytosolic proteins in mesangial cells with 2 h high glucose concentrations (data not shown). Together, increased expression of PSMA2 and decreased expression of PDI with acute exposure to high glucose concentrations suggest regulation of pathways involved in proteostasis and/or cell stress response. In the ER, PDI serves an oxidoreductase chaperone regulating disulfide bonds [43] and its activity is decreased in liver cells of diabetic mice [44]. Furthermore, kidneys and liver of diabetic mice also have decreased expression of PDI [45, 46]. Decreased expression of PDI in response to high glucose may alter protein maturation in the ER, triggering a stress response which includes increased protein degradation by the proteasome. The mechanism of decreased PDI expression in mesangial cells by acute exposure to high glucose remains to be defined.

In conclusion, the proteomics data and bioinformatic data analysis suggests that murine GMCs respond to acute HG via expression of proteins related by pathways regulating protein posttranslational modification and protein stability. These acute differences may also be important for cellular function as reported for GMCs treated with longer more chronic hyperglycemic time points of differences in specific protein abundance such as enolase, actin, and annexin proteins [30].

## Figures and Tables

**Table 1:** 

Spot	Protein name	Gene product	(HG/NG^*∗*^)	Theoretical		Observed		IPA network	Percent coverage
				*M*_*r*_	pI	*M*_*r*_	pI		
1	Not identified		1.23						
2	10 kDa heat-shock protein, mitochondrial	CH10_RAT	2.37	10895	8.9	8000	8.3	n/a	77
3	Calpactin I light chain	S10AA_RAT	1.37	11182	6.3	8000	7.4	1	45
4	Macrophage migration inhibitory factor	MIF_RAT	1.34	12640	6.8	9000	7.8	3	26
5	Cofilin-1	COF1_RAT	1.5	18749	8.2	9000	5.3	n/a	34
6	Not identified		1.70						
7	Not identified		1.24						
8	Profilin-1	PROF1_RAT	1.34	15119	8.5	15000	8.4	1	45
9	Cystatin B	CYTB_RAT	0.82	11303	5.9	10000	6.5	2	80
10	Not identified		0.66						
11	Coactosin-like protein	COTL1_MOUSE	1.74	16048	5.3	16000	5.5	1, 2	37
12	Galectin-1	LEG1_RAT	1.23	15189	5.1	15000	5.3	3	61
13	Histidine triad nucleotide-binding protein 1	HINT1_MOUSE	1.39	13882	6.4	11000	7.3	3	49
14	40S ribosomal protein S12	RS12_RAT	1.34	14858	6.8	15000	7.2	2	44
15	Nucleoside diphosphate kinase A (NDK A)	NDKA_RAT	1.68	17296	6.0	16000	6.6	3	39
16	Nucleoside diphosphate kinase B	NDKB_RAT	1.26	17386	6.9	18000	7.6	1	66
17	Not identified		1.89						
18	Eukaryotic translation initiation factor 5A	IF5A1_RAT	1.23	17049	5.1	20000	5.6	2	43
	MIR-interacting saposin-like protein	MSAP_MOUSE		21096	5.0	20000	5.6	n/a	29
19	Not identified		1.64						
20	Cofilin-1	COF1_RAT	1.63	18749	8.2	21000	8	1	65
21	Cofilin-1	COF1_RAT	1.59	18749	8.2	21000	7.6	1	60
22	Cofilin-1	COF1_RAT	1.57	18749	8.2	21000	7	1	54
23	Not identified		1.61						
24	Proteasome subunit alpha type 1	PSA2_RAT	1.54	26024	6.9	23000	7.5	1	47
25	Heat-shock protein beta-1	HSPB1_RAT	2.31	22936	6.1	23000	6.1	1	39
	Phosphoserine phosphatase	SERB_RAT		25180	5.5	23000	6.1	n/a	33
26	14-3-3 protein epsilon	1433E_RAT	1.37	29326	4.6	30000	4.8	1	29
27	Proteasome subunit alpha type 2	PSA1_RAT	1.78	29784	6.2	31000	7.2	1	36
28	Prohibitin	PHB_RAT	2.24	29859	5.6	32000	6.1	2	62
29	Not identified		2.33						
30	Proliferating cell nuclear antigen	PCNA_RAT	1.46	29072	4.6	34000	4.9	1	31
31	Heat-shock protein beta-1	HSPB1_MOUSE	0.70	23057	6.1	35000	6.3	n/a	25
32	Annexin A2	ANXA2_RAT	0.83	38939	7.6	40000	7.8	1	56
33	Reticulocalbin 3 precursor	RCN3_HUMAN	1.80	37470	4.7	41000	4.9	2	20
34	Macrophage capping protein	CAPG_RAT	1.53	39060	6.1	41000	6.9	3	20
35	Acetyl-CoA acetyltransferase, cytosolic	THIC_RAT	2.15	41538	6.9	41000	7.7	3	28
36	SUMO-activating enzyme subunit 1	SAE1_RAT	2.15	38945	5.0	41000	5.4	2	54
37	Actin, cytoplasmic-1 (beta-actin)	ACTB_RAT	0.58	42052	5.3	42000	5.7	1	30
	Actin, cytoplasmic-2 (gamma-actin)	ACTG_RAT		42108	5.3	42000	5.7	1	30
38	Not identified		0.6						
39	Actin-like protein 3	ARP3_MOUSE	0.53	47783	5.6	50000	6.5	1	38
40	Enolase 1	ENOA_RAT	0.60	47440	6.2	52000	6.7	1	38
	RAB GDP dissociation inhibitor beta	GDIB_RAT		51018	5.9	52000	6.7	n/a	34
41	Not identified		0.37						
42	Not identified		0.71						
43	Protein disulfide-isomerase A3 (ERp57)	PDIA3_RAT	0.78	57044	5.9	58000	6.4	3	50
44	GRP58	HNRPK_RAT	0.68	51230	5.4	57000	6.3	1	31
45	Not identified		2.21						
46	Heterogeneous nuclear ribonucleoprotein L	HNRPL_MOUSE	1.40	60712	6.7	58000	7.8	2	25
47	T-complex protein 1, epsilon subunit	TCPE_RAT	0.78	59955	5.5	58000	6.3	1	34
48	Hsc70/Hsp90-organizing protein	STIP1_RAT	1.16	63158	6.4	65000	7.2	1	38
49	GRP 75	GRP75_RAT	0.82	74097	6.0	75000	6.1	1	31
50	Not identified		1.35						
51	RAB 6 interacting protein 2 (ERC protein 1)	GANAB_MOUSE	0.68	107300	5.7	116000	6.4	2	21

Murine GMC protein expression at 2 h culture HG versus 2 h culture NG^*∗*^.
